# A cross-sectional analysis of HIV and hepatitis C clinical trials 2007 to 2010: the relationship between industry sponsorship and randomized study design

**DOI:** 10.1186/1745-6215-15-31

**Published:** 2014-01-22

**Authors:** Neela D Goswami, Ephraim L Tsalik, Susanna Naggie, William C Miller, John R Horton, Christopher D Pfeiffer, Charles B Hicks

**Affiliations:** 1 Department of Epidemiology, Rollins School of Public Health, Department of Medicine, Emory University School of Medicine, Atlanta, GA, USA; 2 Department of Medicine, Duke University Medical Center, Durham, NC, USA; 3 Duke Clinical Research Institute, Durham, NC, USA; 4 Department of Epidemiology, Gillings School of Public Health, Department of Medicine, University of North Carolina, Chapel Hill, NC, USA; 5 GlaxoSmithKline Health Care, Parsippany, NJ, USA; 6 Department of Hospital and Specialty Medicine, Portland VA Medical Center, Portland, OR, USA; 7 Division of Infectious Diseases, Oregon Health and Science University, Portland, OR, USA; 8 Research Service, Durham VAMC, 27710 Durham, NC, USA

**Keywords:** Industry, Pharmaceutical, Bias, Randomization, Methodology, Trial

## Abstract

**Background:**

The proportion of clinical research sponsored by industry will likely continue to expand as federal funds for academic research decreases, particularly in the fields of HIV/AIDS and hepatitis C (HCV). While HIV and HCV continue to burden the US population, insufficient data exists as to how industry sponsorship affects clinical trials involving these infectious diseases. Debate exists about whether pharmaceutical companies undertake more market-driven research practices to promote therapeutics, or instead conduct more rigorous trials than their non-industry counterparts because of increased resources and scrutiny. The ClinicalTrials.gov registry, which allows investigators to fulfill a federal mandate for public trial registration, provides an opportunity for critical evaluation of study designs for industry-sponsored trials, independent of publication status. As part of a large public policy effort, the Clinical Trials Transformation Initiative (CTTI) recently transformed the ClinicalTrials.gov registry into a searchable dataset to facilitate research on clinical trials themselves.

**Methods:**

We conducted a cross-sectional analysis of 477 HIV and HCV drug treatment trials, registered with ClinicalTrials.gov from 1 October 2007 to 27 September 2010, to study the relationship of study sponsorship with randomized study design. The likelihood of using randomization given industry (versus non-industry) sponsorship was reported with prevalence ratios (PR). PRs were estimated using crude and stratified tabular analysis and Poisson regression adjusting for presence of a data monitoring committee, enrollment size, study phase, number of study sites, inclusion of foreign study sites, exclusion of persons older than age 65, and disease condition.

**Results:**

The crude PR was 1.17 (95% CI 0.94, 1.45). Adjusted Poisson models produced a PR of 1.13 (95% CI 0.82, 1.56). There was a trend toward mild effect measure modification by study phase, but this was not statistically significant. In stratified tabular analysis the adjusted PR was 1.14 (95% CI 0.78, 1.68) among phase 2/3 trials and 1.06 (95% CI 0.50, 2.22) among phase 4 trials.

**Conclusions:**

No significant relationship was found between industry sponsorship and use of randomization in trial design in this cross-sectional study. Prospective studies evaluating other aspects of trial design may shed further light on the relationship between industry sponsorship and appropriate trial methodology.

## Background

Over the past 15 years, the proportion of clinical research sponsored by the pharmaceutical industry has increased and likely will continue to expand as federally funded research at academic centers faces massive cuts [1]. In 2000, 70% of the clinical drug trial enterprise was funded by industry rather than by the National Institutes of Health (NIH), and as of 2010 this proportion is increasing [2,3]. Pharmaceutical companies fill a critical need by providing substantial upfront investment in the drug development process. This is particularly evident with chronic diseases that may have a low event rate over a long period of time. It is, however, axiomatic that business interests are a primary consideration in this process, and generation of profits over time is thus a central necessary theme. This may not always align with the goal of identifying the most effective treatments that maximize public health [4,5].

Ross and colleagues document some techniques that industry has used to tilt the research framework in their favor, including seeding trials, inappropriate authorship, market-driven publication planning, and selective publication and reporting [6].

The role of industry in HIV trials is particularly interesting as HIV has evolved into a chronic comorbidity, rather than a death sentence, with high profits for manufacturers of lifelong, well-tolerated antiretrovirals. Hepatitis C (HCV) has also recently attracted attention from industry; in the past decade, three major anti-HCV agents have gone through the Food and Drug Administration (FDA) submission process, supported by trials with sponsorship from both industry and academic-based groups. A scarcity of data exists, however, on the potential influence of industry sponsorship on infectious diseases (ID) studies, given that industry has traditionally been less likely than government to conduct HIV and other virus-related research due to the public health, rather than market-driven, nature of these infections [7,8].

Prior epidemiology studies have compared several characteristics of general industry-sponsored versus non-industry-sponsored trials, including the following: (1) methods of subject recruitment [5]; (2) outcomes favoring the investigational drug [9-13]; (3) reporting and publication timing of outcomes and adverse drug events [5,6,12,14-16]; (4) affiliations of publication authors [12,17]; and (5) reasons for early study termination [11,18,19]. These studies, however, have generally been limited to the published literature, sometimes to a few core journals, rather than evaluating all trials registered in a public database, and therefore may not be capturing the full extent of potential industry-related bias. While limited data exists for ID, and HIV and HCV trials in particular, recent data suggests sponsors of industry-funded ID trials are more likely to add, omit, or reclassify outcomes compared to other specialties, and are more susceptible to publication bias [20,21]. ID trials have been less studied, in part because there are not dedicated ID trial registries, and also because some ID fields, such as HIV and HCV, have received only intermittent waves of new promising therapies. This differs from the field of oncology, where new agents are constantly undergoing evaluation by the FDA, and potential differences in methodology between trials based on trial sponsorship have been extensively scrutinized [19].

The aim of this study was to evaluate if industry-sponsored HIV/AIDS and HCV drug intervention trials entered in a public registry were less likely to use a randomized study design than non-industry-sponsored trials, after adjustment for presence of a data monitoring committee, anticipated or actual enrollment, number of study sites, inclusion of foreign study sites, exclusion of persons older than age 65, and disease condition. We also evaluated if this relationship differed based on study phase since phase 4 trials, generally conducted after FDA drug approval, may be less influenced by federal regulations than phase 2 and 3 trials.

## Methods

In this cross-sectional study, we analyzed data from trials registered in the ClinicalTrials.gov registry from 2007 through 2010 to study the association between industry sponsorship of interventional trials and randomized study design.

### Data source

To address concerns related to study design and publication bias, the FDA mandated public registration of all trials of drugs, biologics, or devices intended for FDA submission with penalties for non-compliance in 2007. The International Committee of Medical Journal Editors (ICMJE) also implemented a policy requiring prospective registration of phase 2 to 4 trials for publication in member journals. ClinicalTrials.gov is the largest US-based database of its kind that allows investigators to fulfill these mandates and covers a full range of clinical conditions as well as a broad group of trial sponsors [22]. Transformation of the registry database into a research dataset (Aggregate Analysis of ClinicalTrials.gov) by the Clinical Trials Transformation Initiative (CTTI), a collaboration between the FDA and Duke University, provides the opportunity for critical evaluation of study designs for industry-sponsored trials, independent of publication status. The methods used by ClinicalTrials.gov to register clinical trials have been described previously [22]. Briefly, trial sponsors and investigators from around the world can enter trial data through a web-based data entry system.

The ID interventional clinical trials sub-dataset was created in 2011 to describe trial methodology, geographic distribution, and funding source of current ID trials [23] and includes 3,570 trials registered with ClinicalTrials.gov from 1 October 2007 to 27 September 2010. As part of this study, trials were subcategorized into content areas based on study title and description. A total of 58 categories were defined, including HIV-AIDS; HCV; lower respiratory tract infection (LRTI); hepatitis B; malaria; diarrheal diseases; sexually transmitted diseases (STD) excluding HIV; tuberculosis; childhood cluster diseases; and meningitis.

### Study design

**Figure 1:**
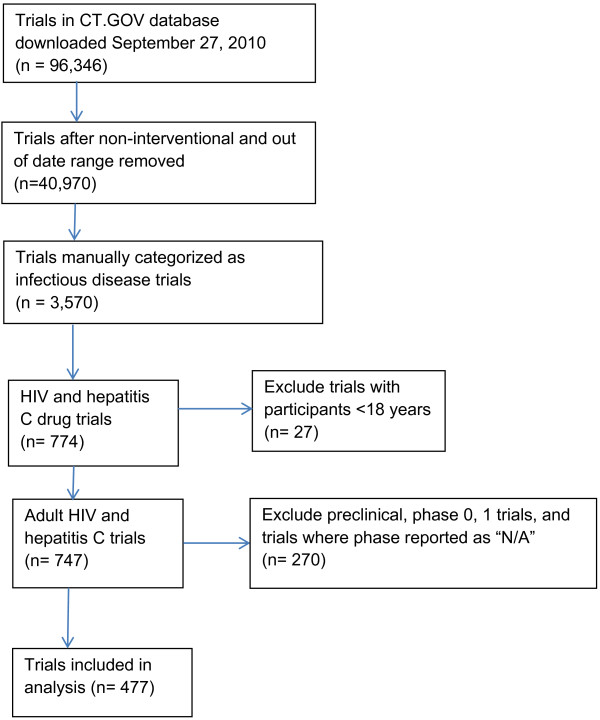
**Trial selection from the ClinicalTrials.gov registry, 2007 to 2010.** A sub-group of 774 HIV and hepatitis C virus (HCV) drug intervention clinical trials was identified for cross-sectional analysis.

From the ID dataset, we conducted a cross-sectional analysis of 774 HIV and HCV drug intervention clinical trials. Interventional trials involving a procedure, vaccine, behavioral intervention, or device were not included. Since our target analysis was for adult-only trials, we excluded trials that included children (age less than 18 years old). We excluded all nonhuman preclinical trials, phase 0 pharmacokinetic/pharmacodynamics trials, and phase 1, phase 1/2 dose escalation studies, as these by definition are single arm studies without a randomized study design (Figure 1). Studies in which a phase was reported as ‘Not applicable’ were also excluded.

### Variable definitions

The primary exposure, industry sponsorship, was derived from lead sponsor, a nominal categorical variable reported by trial investigators and coded dichotomously. In the web-based data entry system, lead sponsor was described as the primary organization that oversees study implementation and is responsible for conducting data analysis. Outcome was randomized (versus non-randomized) study design and was reported by sponsor on the ClinicalTrials.gov data entry website. Randomization was chosen as the outcome since choice of this design would be most likely to yield unbiased results. Based on prior studies of registry clinical trials and potential associations with the exposure and outcome, the following covariates were analyzed: trial status, enrollment sample size (anticipated or actual), study start year, study phase, presence of a data monitoring committee, exclusion of persons older than age 65, number of study sites, disease condition, and inclusion of foreign study sites.

For active studies that had not completed recruitment, data entrants recorded anticipated enrollment. For studies that had completed or terminated recruitment, actual enrollment was reported. Enrollment was reported in the original dataset as a continuous variable. Based on prior literature describing trial size in pre-specified categories [22], data was assigned into the following categories: less than or equal to 100, 101 to 1,000, and greater than 1,000 participants. Study start year was available in the original dataset for both future trials (anticipated start year) and completed trials (actual start year). As our analysis focused on the 2007 to 2010 time frame, earlier studies were consolidated into a ‘Prior to 2007’ category and values for the remaining observations were maintained. Location of study facilities (sites) was defined by the study sponsor and could include more than one location for multi-site studies. This information was used to determine if the study included non-US sites. Number of study sites was reported as multi-site versus single site.

### Statistical analysis

Summary statistics were calculated by pooling across active and completed studies. To control for multiple variables simultaneously, we carried out multivariable Poisson regression analysis to calculate prevalence ratio (PR) estimates. These models included industry sponsorship as the primary exposure, randomization use as the outcome, and all factors statistically associated either in the literature or in our univariate models with randomized study design.

Effect measure modification was assessed by using a likelihood ratio (LR) test to compare the fully-adjusted model (which included the product term for industry sponsorship by study phase) to a nested model with the product term removed. An *a priori* criterion of *P* < 0.15 was used to determine whether it was necessary to include the interaction term. Colinearity of similar variables was evaluated with Pearson’s correlation testing; for variables with a Pearson’s coefficient (r) ≥ 0.7, only the more relevant variable was included. SAS software, version 9.2 (SAS Institute, Cary, NC, USA) was used for all statistical analyses.

### Power calculation

The study required a sample of 366 trials to achieve 80% power, assuming that 65% of registered trials from non-industry sponsors would use randomization and 50% from industry sponsors.

### Procedures for the ethical conduct of research

The protocol for this study was reviewed and declared as exempt by the University of North Carolina (UNC) School of Public Health Institutional Review Board (IRB), Protocol Number 119879.

## Results

A total of 477 HIV and/or HCV adult interventional clinical drug trials were included, with 304 (64%) HIV/AIDS trials, 156 (33%) HCV trials, and 17 (4%) co-infection trials. Trial characteristics are shown in Table 1. Our sample included 179 (38%) industry-sponsored trials and 298 (63%) non-industry-sponsored trials. Most non-industry trials were sponsored by academic institutions or hospitals (Table 2). Of the 469 trials with reported allocation data, 329 (69%) had a randomized study design and 140 (29%) were non-randomized. The majority of studies were actively recruiting or had completed recruitment of participants (n = 300, 63%), while the remainder either were not recruiting at the time of analysis (n = 164, 34%) or had undergone study termination (n = 13, 3%). Most interventional HIV and HCV trials were small, with a median anticipated or actual sample size of 80 persons (interquartile range (IQR): 36, 179), and 288 (60%) trials with 100 participants or less.

**Table 1:** Characteristics of eligible HIV/Hepatitis C interventional trials, ClinicalTrials.gov registry, 2007 to 2010 (N = 477)

**Characteristic**	**N**	**% or IQR**
Primary Sponsor		
Non-industry	298	62.5
Industry	179	37.5
Allocation^a^		
Non-Randomized	140	29.4
Randomized	329	69.0
Overall status		
Not yet recruiting	41	8.6
Recruiting	206	43.2
Active not currently recruiting	123	25.8
Completed	94	19.7
Terminated	13	2.7
Number of participants^a^		
Median (IQR)	80	36, 179
Less than or equal to 100	288	60.4
101 to 1,000	173	36.3
Greater than 1,000	14	2.9
Start year^a^		
Before 2007	63	13.2
2007	42	8.8
2008	130	27.3
2009	137	28.7
2010	98	20.6
Phase		
2/3	284	59.5
4	193	40.5
Study has DMC^ab^		
No	207	43.4
Yes	189	39.6
Excludes > 65 years		
No	352	73.8
Yes	125	26.2
Number of study sites^a^		
Single facility	205	43.0
Multiple facilities	225	47.2
Foreign sites		
No	126	26.4
Yes	351	73.6
Condition		
Hepatitis C (HCV)	156	32.7
HIV/AIDS	304	63.7
HCV + HIV/AIDS	17	3.6

^a^Totals for each variable may not add up to 100 % secondary to observations with missing data.

^b^DMC = data monitoring committee. IQR = interquartile range.

**Table 2:** Sponsors of eligible HIV/Hepatitis C interventional trials, ClinicalTrials.gov registry, 2007 to 2010 (N = 477)

**Sponsor**	**N**	**%**
Industry	179	37.5
NIH	18	3.8
US Federal	5	1.1
Government-Foreign	24	5.0
Academic/Hospital	179	37.5
Consortium	30	6.3
Other	72	8.8

This dataset focused on trials registered between 2007 to 2010, and most studies had a start date of 2008 or later; only 63 (13%) started before 2007. Overall, more studies were phase 2 or 3 (n = 284, 60%) rather than phase 4 (n = 193, 41%) and about 40% (n = 189) utilized a data monitoring committee (DMC). Exclusion of elderly (persons older than 65 years old) was uncommon in our studies; 352 (74%) of studies did not report this as an exclusion criteria. A similar proportion of studies were single-site studies (43%, n = 205) as compared to those that included multiple sites for recruitment of participants (47%, n = 225). All interventional trials with at least one US site are required by the FDA to be reported in a public registry, [22] but most HIV/hepatitis studies (n = 351, 74%) also included foreign sites.

All variables had fewer than 15% observations with missing data in this dataset except for reporting of a DMC, for which 81 trials (17%) had a missing value. In a multivariable predictive model for missing DMC data, missing was not associated with use of randomization, actual/anticipated enrollment, study phase, trial exclusion of elderly persons, number of sites, inclusion of foreign study sites, or disease condition. Trials missing DMC data were more likely to be industry-sponsored (PR = 4.03, 95% CI 2.05, 7.92) than trials not missing data on use of a DMC.

Effect measure modification was not found to be statistically significant by study phase (phase 2/3 versus phase 4, *P* = 0.30. In stratified tabular analysis the adjusted PR was 1.14 (95% CI 0.78, 1.68) among phase 2/3 trials and 1.06 (95% CI 0.50, 2.22) among phase 4 trials. Interaction was also not significant for disease condition (HIV/AIDS versus HCV, *P* = 0.84), or use of a DMC (*P* = 0.54).

We found sample size, study phase, number of study sites, inclusion of foreign sites, and disease condition were associated with industry sponsorship (Table 3). Industry-sponsored trials were more likely to have large sample sizes (100 to 1,000 participants) than small sample sizes (less than or equal to 100 participants, PR = 1.76, 95% CI 1.31, 2.38), multi-center rather than single center studies (PR = 4.25, 95% CI 2.74, 6.61), and include foreign sites (PR = 2.21, 95% CI 1.45, 3.37). Industry trials were less likely to be HIV/AIDS trials than HCV trials (PR = 0.39, 95% CI 0.29, 0.53) and also less likely to be phase 4 trials than phase 2 or 3 (PR = 0.25, 95% CI 0.16, 0.38). The covariates we analyzed were not significantly associated with use of randomized study design in this sample (data not shown).

**Table 3:** Association between trial characteristics and industry sponsorship in eligible HIV/Hepatitis C interventional trials, ClinicalTrials.gov registry, 2007 to 2010 (N = 477)

**Characteristic**	**Industry-sponsored**	**Non-industry-sponsored**	**PR**^**c**^	**95% CI**
Number of participants^a^				
Median (IQR)	120 (50,290)	60 (30,140)		
Less than or equal to 100	84	204	1.0	
101 to 1,000	89	84	1.76	1.31, 2.38
Greater than 1,000	5	9	1.22	0.50, 3.02
Start year^a^				
Before 2007	24	39	1.0	
2007	16	26	1.0	0.53, 1.88
2008	55	75	1.11	0.69, 1.79
2009	52	85	1.0	0.61, 1.62
2010	30	68	0.80	0.47, 1.37
Phase				
2/3	153	131	1.0	
4	26	167	0.25	0.16, 0.38
Study has DMC^ab^				
No	69	138	1.0	
Yes	52	137	0.83	0.58, 1.18
Excludes > 65 years				
No	128	224	1.0	
Yes	51	74	1.12	0.81, 1.55
Number of study sites^a^				
Single facility	24	181	1.0	
Multiple facilities	112	113	4.25	2.74, 6.61
Foreign sites				
No	25	101	1.0	
Yes	154	197	2.21	1.45, 3.37
Condition				
Hepatitis C (HCV)	99	57	1.0	
HIV/AIDS	76	228	0.39	0.29, 0.53
HCV + HIV/AIDS	4	13	0.37	0.14, 1.01

^a^Totals for each variable may not add up to N = 468 secondary to observations with missing data.

^b^DMC = data monitoring committee.

^c^PR = unadjusted prevalence ratio, calculated from Poisson regression model with covariate as exposure and industry sponsorship as outcome.

IQR = interquartile range.

We found that use of randomization was as likely in registered industry-sponsored trials as in non-industry-sponsoreded trials (Table 4, PR 1.17, 95% CI 0.94, 1.45). This persisted even after adjusting for other characteristics associated with choice of randomized study design, including presence of a DMC, study phase, sample size, number of study sites, inclusion of foreign study sites, exclusion of persons older than age 65 and disease condition (PR = 1.13, 95% CI 0.82, 1.56).

**Table 4:** Unadjusted and adjusted prevalence ratios for use of randomization with industry and non-industry sponsors in eligible HIV/Hepatitis C interventional trials, ClinicalTrials.gov registry, 2007 to 2010 (N = 468)

**Sponsor**	**Prevalence ratio**	**95% CI**
Unadjusted:	1.17	0.94, 1.45
Industry sponsor		
Adjusted:^a^	1.13	0.82, 1.56
Industry sponsor		

^a^Based on Poisson regression model, adjusted for presence of a data monitoring committee, enrollment size, study phase, number of study sites, inclusion of foreign study sites, exclusion of persons older than age 65, and disease condition.

## Discussion

Our data suggests that in registered HIV and HCV trials, regardless of publication, randomization is not significantly differentially utilized by industry, and is not a clear manifestation of industry bias. The goal of mandated public trial registration is to reduce bias by allowing increased critical evaluation of trial design and to encourage full outcome reporting. Our analysis is one of a recent few that have looked at trends in registered trials to see if registration may be meeting this goal, facilitated by the CTTI-initiated ClinicalTrials.gov transformation into a research dataset. While some authors suggest industry trials may manipulate study methodology to report a desired outcome or achieve other goals, other academics suggest that industry trials may actually have the same or higher standards for methodological quality because of closer scrutiny [5,11,24,25].

Randomized clinical trials are the ‘gold standard’ for clinical research, but there are ways *within* randomized trials to introduce bias, and this should be further scrutinized in comparing ID trials with different sponsors. Previous authors have evaluated the use of blinding, selection of sample size, choice of comparator, analytic methods, partial versus full reporting of outcomes, and agreement between results and conclusions in industry-sponsored trials, providing some evidence for industry bias [6,14,26-28]. Lathyris, *et al*. for example, found that companies are more likely to choose only their own products as comparators rather than conducting more medically appropriate head-to-head trials with drugs from different companies [26], and other studies describe pharmaceuticals choosing placebo or a suboptimal agent when an effective comparator exists [27].

Randomized HIV and HCV trials may also not be the best option for some situations where the number of patients under study is too few and other designs, such as crossover trial designs, are more appropriate. Randomization is furthermore not essential in situations of clinical ‘equipoise’, when the optimal standard of care is unclear. Study design is also only one step in the clinical trial inception and implementation cascade, and there are multiple other points of potential bias introduction. We chose to focus on randomization as a methodologic choice, but prior studies have shown that sponsor bias may influence other downstream steps in the process [5,6,10,12-14]. The most relevant final action susceptible to sponsor bias is physicians’ use of evidence-based medicine after study publication, which has been the major target of the Patient Protection and Affordable Care Act’s Physician Payment Sunshine provision [6].

The major limitations to our study were the fact that all trial data was self-reported by study sponsors, rather than determined by an objective third party, and that unregistered trials were not included, similar to other analyses using the ClinicalTrials.gov database [29]. The registry also does not provide a means to assess the strength of the randomization process in any study. We furthermore measured industry involvement in trials by sponsorship, rather than by industry funding or author affiliation with industry, given that the latter are not directly reported in ClinicalTrials.gov. Given that many non-industry-sponsored trials may receive industry funding at some level and therefore be susceptible to some amount of industry bias, it is possible that we underestimated the association between industry involvement and use of randomization.

Our study also focused on trials registered with ClinicalTrials.gov, one of several registries where trials can be reported, including the International Standard Randomized Control Trial Number Register (http://isrctn.org), World Health Organization (WHO) International Clinical Trials Registry (http://www.who.int/trialsearch/), and corporate trial registries and databases of manufacturers of drugs. ClinicalTrials.gov is the largest US-based registry, however, and may be generalizable for all registered HIV and HCV interventional trials.

Given that trials cannot be registered without completion of all mandatory data elements and are required to conform to relevant national health regulations, we had few missing data. For the one variable that did have a significant percentage of missing data, utilization of a data monitoring committee, our analysis showed this was more likely to go unreported in industry- sponsored trials. This could be explained by other arrangements for safety monitoring in industry trials besides the use of DMC, such as use of a contract ethics review board.

We expected to see a stronger association of industry sponsorship and decreased use of randomization in phase 4 HIV and HCV trials (compared to phase 2 and 3 trials), since these are conducted after FDA drug approval and therefore generally have less governmental oversight. Phase 4 registered trials on ClinicalTrials.gov have also been shown previously to report less use of blinding and randomization overall [22]. Effect measure modification was not significant in our analysis, however. This could be due to the fact that post-registration HIV/AIDS and HCV trials sponsored by industry are more likely to collaborate with academic institutions and consortia than other disease conditions, or are under more public scrutiny overall.

## Conclusions

In this cross-sectional analysis of publicly registered HIV/AIDS and HCV adult phase 2 to 4 interventional clinical trials, we found randomized study design was as likely in industry-sponsored trials as in non-industry-sponsored trials. The potential for industry bias in the full cascade of publicly registered trials, from study design to implementation and presentation, though, requires further critical evaluation.

## Abbreviations

CTTI: Clinical Trials Transformation Initiative; DMC: data monitoring committee; FDA: Food and Drug Administration; HCV: hepatitis C virus; HIV: human immunodeficiency virus; ICMJE: International Committee of Medical Journal Editors; ID: infectious diseases; IQR: interquartile range; LRTI: lower respiratory tract infection; NIH: National Institute of Health; PR: prevalence Ratio; STD: sexually transmitted disease; WHO: World Health Organization.

## Competing interests

CBH has research funded by Argos, BMS, Gilead, Viiv, and Merck and serves on the scientific advisory committee for BMS, Gilead, Merck, Viiv, and Jansen Virology. SN serves on an advisory board for Vertex and Boehringer Ingelheim and receives grant support from Vertex, Anadys, Synexis, BMS, and Medtronic.

## Authors’ contributions

The study was conceived by NDG, ELT, CBH and SN. These authors, along with WCM and JRH participated in study design and analysis. ELT, JRH, NDG and CDP collected and interpreted data for this study. NDG drafted the manuscript, and ELT, SN, WCM, and CBH made critical revisions. All authors reviewed and approved the final manuscript.
